# New insights into the impacts of suspended particulate matter on phytoplankton density in a tributary of the Three Gorges Reservoir, China

**DOI:** 10.1038/s41598-017-13235-0

**Published:** 2017-10-18

**Authors:** Qiang He, Yixi Qiu, Haohang Liu, Xingfu Sun, Li Kang, Li Cao, Hong Li, Hainan Ai

**Affiliations:** 1 Key Laboratory of Eco-Environment of Three Gorges Region, Ministry of Education, Chongqing University, Chongqing, 400044 China; 2 Xiamen Municipal Engineering Design Institute CO., LTD, Chongqing, 401122 China; 3 Chongqing Green Environment Protection Technology CO., LTD, Chongqing, 400045 China

## Abstract

Phytoplankton density can be influenced by a wide range of factors whereas the role of suspended particulate matter (SPM) are not clear in river that annually subjected to hydrodynamics shift. Here, spatial-temporal variation of environmental parameters and phytoplankton density were studied from January 2013 to December 2014 in Yulin River, a tributary of the Three Gorges Reservoir, China. Laboratory experiments were conducted to elucidate the key parameter and interpret how it impacted phytoplankton density. SPM is negatively correlated with phytoplankton density. Despite SPM in Yulin River revealed weaker NH_3_-N, NO_3_-N and PO_4_-P adsorption capabilities in comparison to that in other aquatic ecosystems, increase of water velocity from 0.1 to 0.8 m/s led to approximately 6.8-times increase of light attenuation rate. In experiments evaluating the aggregation of *Chlorella pyrenoidosa* upon SPM, floc size showed 7.4 to 22% fold increase compared to the SPM or algae itself, which was due to the interaction between SPM and phytoplankton extracellular polymeric substances. Our results suggest that SPM could contribute to the variation of phytoplankton density through the integrated process including light attenuation, nutrient adsorption and algae aggregation. This is the first evaluation of the multiple processes underlying the impact of SPM on phytoplankton.

## Introduction

Phytoplankton communities are key components in determining ecosystem stability in aquatic environments and contributes nearly half of global primary production. Studies on the proliferation phytoplankton over the last decade had indicated a wide range of factors that could influence the phytoplankton communities or densities. It is now widely accepted that the flourishing of phytoplankton densities in aquatic environment was the result of increasing nutrients levels associated with a variety of environmental factors, including low water turbulence, low light, low ratio of euphotic zone to mixing zone and high temperature, among which the dominant factor governing the growth of phytoplankton is the availability of mineral and/or organic nutrients^1^. Although the role of nitrogen (N), phosphorus (P), or NP ratio (N/P) in phytoplankton cell growth in still a matter of debate^2^, it is most likely that the availability of dissolved inorganic N limits phytoplankton growth, whereas in some cases, P availability plays a key role^3^. Light conditions are critically important both in terms of‘ quantity and quality (wavelength) and can significantly impact the proliferation of planktonic algae.

The above mentioned factors are universal throughout lakes, rivers, and reservoirs. However, in river ecosystems that subjected to seasonal hydrologic alterations, spatial and temporal variability in the distribution of suspended particulate matter (SPM) is relatively high, which may influence the density and distribution of phytoplankton. Previously, it was assumed that SPM served as a source or sink of carbon, nitrogen, and phosphorus nutrients and could impacted the phytoplankton communities through affecting the physical and biogeochemical nutrient transport. It was also found that P could be absorbed on the SPM and further mitigated into the sediment layers while increasing the mobile P content in water column^4^, which may favor phytoplankton growth. This was supported by the outcomes of studies regarding the nutrient transports in Huanghe basin, China^5^.

The damming of rivers to create reservoirs not only affect the physical, chemical, and biological characteristics of the streamflow^6^, but also produces more suitable conditions for phytoplankton growth and substantially alters phytoplankton species assemblages^7^. The Three Gorges Project (TGP) on the Yangtze River, China, has been a focus of international interest because of its potential and far-reaching effects on the ecosystem. Since the initial filling of the Three Gorges Reservoir (TGR) in June 2003, algal blooms begin to occur annually in the reservoir and in some upstream tributaries, and intense phytoplankton blooms have been observed in some tributary backwaters even in winter^8^. Both the frequency of phytoplankton blooms and the numbers of tributaries involved have increased as the normal high water level has shifted upwards.

High levels of nutrients in the water could be regulated by SPM, which adsorbs nutrients and therefore inhibits eutrophication. However, studies have shown that SPM may also desorb nutrients, thus facilitating eutrophication. Quantitative assessments could show strong evidence for this. At an SPM concentration of 10 mg/L, typically 20% of the nutrients are in particulate form in estuarine waters, this fraction increases to 60% at an SPM value of 100 mg/L and to 80% at a value of 1,000 mg/L^9^. In the TGR, which is subjected to impounding and discharging on an annual basis, SPM levels on the tributaries generally range from 0.75 to 52.3 mg/L^10^, leading to significant differences in nutrient budgets. Previous studies have revealed that wind-induced and tidal currents can cause frequent sediment re-suspension, resulting in high SPM values of the water column^10^ and, consequently, increased light attenuation, which inhibits phytoplankton growth^1^. Further, a recent study has shown the interaction between SPM and algae extracellular polymeric substance (EPS) could also influence the aggregation property of inorganic particles^11^, which could probably impact the distribution of phytoplankton. To date, although a number of studies have evaluated SPM levels in shallow lakes, little information is available on the impact of SPM on the density of phytoplankton in river-type reservoirs as well as on the underlying mechanism.

In this study, we assessed the spatial and temporal variation of nutrients, phytoplankton density and SPM in the Yulin River, a tributary of the Three Gorges Reservoir, China, during two consecutive years. The environmental factors which influenced the density of phytoplankton were analyzed using principal components analysis (PCA). Subsequently, we evaluated the mechanisms underlying the phytoplankton density upon SPM under laboratory conditions. The overall aim of this study is to fill the knowledge gaps on how SPM influences the density of phytoplankton in SPM-contined river.

## Results

**Figure 1:**
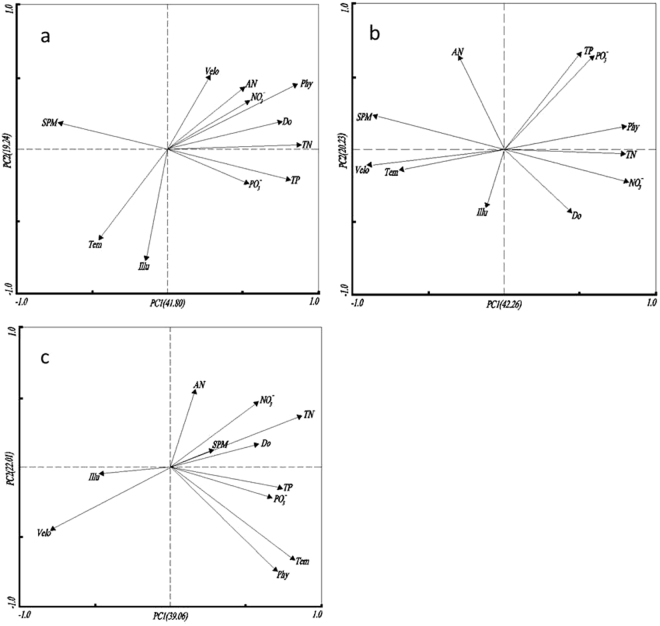
Principal component analyses plot (biplot) displaying the correlation between environmental variables (10 variables) and phytoplankton abundance in sampling site (**a**) Estuary, (**b**) Paihua and (**c**) Shujia.

Impact of environmental factors on phytoplankton density. The nutrients level showed annual response to the TGR dam operations. The TP concentration showed maximum 10-fold increase when the TGR altered from flood season to dry season, in addition, the dissolved PO_4_-P contributed little to TP concentration, indicating TP may predominately maintained in suspended particles. The concentration of TN in the flood season was significantly higher than that in the dry season over the span of the two years. Obviously, the concentration of NO_3_-N was much higher than NH_3_-N. (Table S1). The water velocity of Yulin river showed obvious spatial-temporal variation in response TGR operations. During the flood seasons, the water velocity showed dramatic increase and it was significantly higher at Estuary sites than others. During the initial impounding periods, the Yangzte river may even intrude into Yulin River (Fig. S1). PCA analysis revealed that in the Estuary, Paihua and Shujia sites, two significant components (eigenvalue >1) were extracted, explaining approximately 61.03, 62.48, and 61.07% of the total variance, respectively (Fig. 1a–c). The correlation matrix showed that in the estuary, phytoplankton density was strongly correlated with TN, NO_3_-N, PO_4_-P, DO, TP, NH_3_-N, velocity (ordination axis 1), SPM, temperature, and illumination (ordination axis 2) (Fig. 1a). Pearsons’ correlation coefficient analysis indicated that phytoplankton (Phy in short) density was positively correlated with TN, TP, and DO of the first principal component (PC1) and negatively correlated with SPM (P < 0.05) of the second principal component (PC2). Similarly, in Paihua, phytoplankton abundance was strongly correlated with TN, NO_3_-N, PO_4_-P, DO, and TP (ordination axis 1) and with SPM, velocity, temperature, NH_3_-N as well as illumination (ordination axis 2) (Fig. 1b). Pearson’s correlation coefficient analysis indicated that phytoplankton density was positively correlated with TN and NO_3_-N of the first principal component (PC1) and negatively correlated with SPM (P = −0.608, <0.05) of the second principal component (PC2). In Shujia, phytoplankton density was positively correlated with TN, TP, and temperature of the first principal component (PC1) and negatively correlated with velocity (<0.05) of the second principal component (PC2) (Fig. 1c).

**Figure 2:**
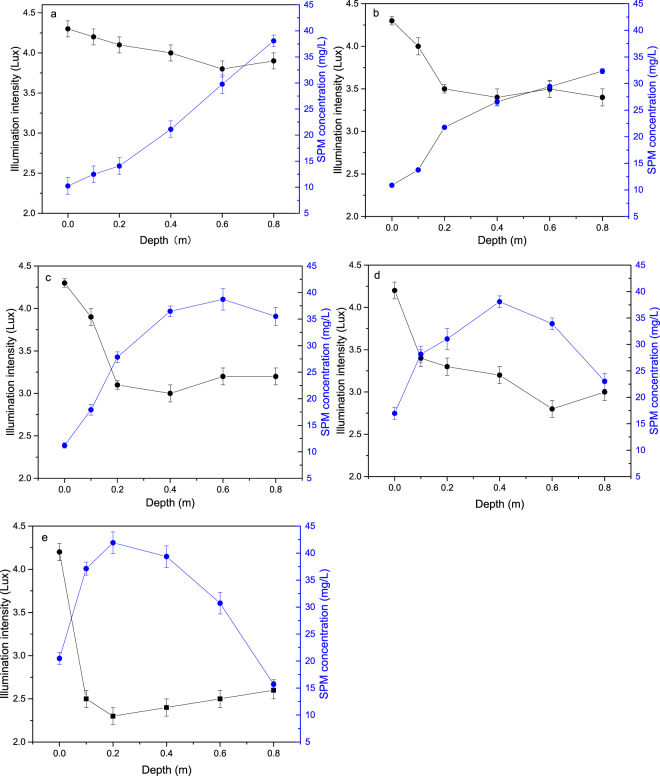
Variation in illumination intensity and SPM concentration under water velocity of (**a**) 0.1 m/s, (**b**) 0.2 m/s, (**c**) 0.4 m/s, (**d**) 0.6 m/s, (**e**) 0.8 m/s. The black dots indicate the illumination intensity (lux) while the blue dots suggest the SPM concentration (mg/L).

Effect of SPM on light attenuation under varied water velocity. With increased water velocity, light attenuation was substantially enhanced. Illumination intensity showed an abrupt decrease with depth and remained at approximately 3.9 lux from 0.6 m downwards when the water velocity maintained at 0.1 m/s (Fig. 2a). When water velocity reached 0.2 m/s, not only the light attenuation rate was prompted, but illumination intensity was also significantly reduced (Fig. 2b). The same pattern was observed when water velocity ranged from 0.4 to 0.8 m/s (Fig. 2c–e), with the minimum illumination intensity of 2.3 lux at 0.2 m below the water surface. With the increase of water velocity, the concentration of SPM varied according to the illumination intensity, with a significant positive relationship (P < 0.05). Based on the data shown in Fig. 2, we conclude that the increase of water velocity led to an enhanced SPM concentration in the surface water, which was responsible for the aggravation of light attenuation.

**Figure 3:**
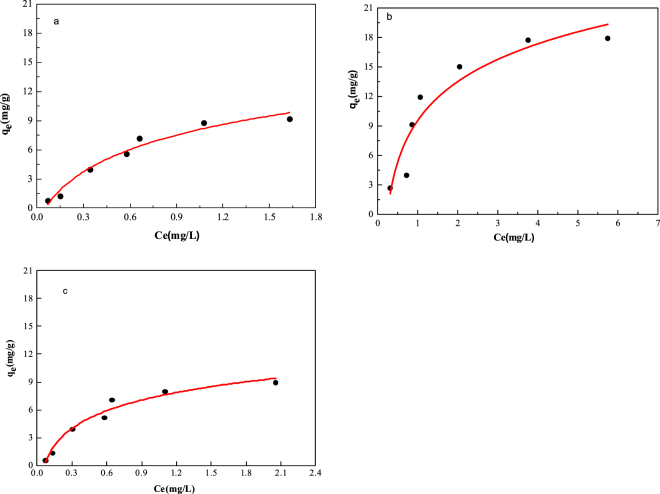
Adsorption isotherms of NH_3_-N (**a**), NO_3_-N (**b**) and PO_4_-P (**c**) in the SPM. q_m_ (mg/g) denotes the maximum adsorption capacity of nutrients, Ce(mg/L) are the equilibrium liquid-phase concentrations of nutrients.

Nutrient adsorption on SPM particles. The sorption isotherms were nonlinear over the range of nutrient concentrations tested (Fig. 3). In both cases, the slope of the isotherm was relatively steep at low nutrient concentrations and approached a plateau at higher concentrations. The difference in the adsorption of the three nutrients was statistically significant (p < 0.05). The nutrient NO_3_-N (Fig. 3b) was adsorbed significantly higher than NH_3_-N (Fig. 3a) and PO_4_-P (Fig. 3c), with maximum adsorption capacities of 9.2, 17.9, and 8.95 mg/g, respectively.

The maximum adsorption capacities (Qmax) for the Langmuir isotherms slightly changed (from 0.83 to 0.88 mg/L, Table 1). The adsorption data for NH_3_-N, NO_3_-N, and PO_4_-P fitted better with the Freundlich isotherms (correlation coefficients R^2^ > 0.88) as compared to the Langmuir isotherms (correlation coefficients R^2^ ≤ 0.80). Adsorption parameters and regression data obtained by Freundlich and Langmuir isotherms demonstrated that NH_3_-N, NO_3_-N, and PO_4_-P have similar adsorption behaviors. Non-linear adsorption was observed for all three nutrients, with K_f_ values ranging from 0.60 to 0.90 mg/L and K_l_ values from 0.037 to 0.063 mg/L. For NH_3_-N and PO_4_-P, we observed the highest degrees of Freundlich and Langmuir isotherms, respectively.

**Table 1:** Sorption coefficients according to Freundlich and Langmuir isotherms for NH_3_-N, NO_3_-N and PO_4_-P.

	Langmuir			Freundlich		
	K_1_ (mg/L)	Q_max_ (mg/kg)	R^2^	k_f_ (mg/L)	N	R^2^
NH_3_-N	0.0412	0.0821	0.4571	0.9026	0.46	0.9404
NO_3_-N	0.0373	0.0880	0.7952	0.6049	0.48	0.8811
PO_4_-P	0.0627	0.0835	0.7363	0.8360	0.51	0.9132

**Figure 4:**
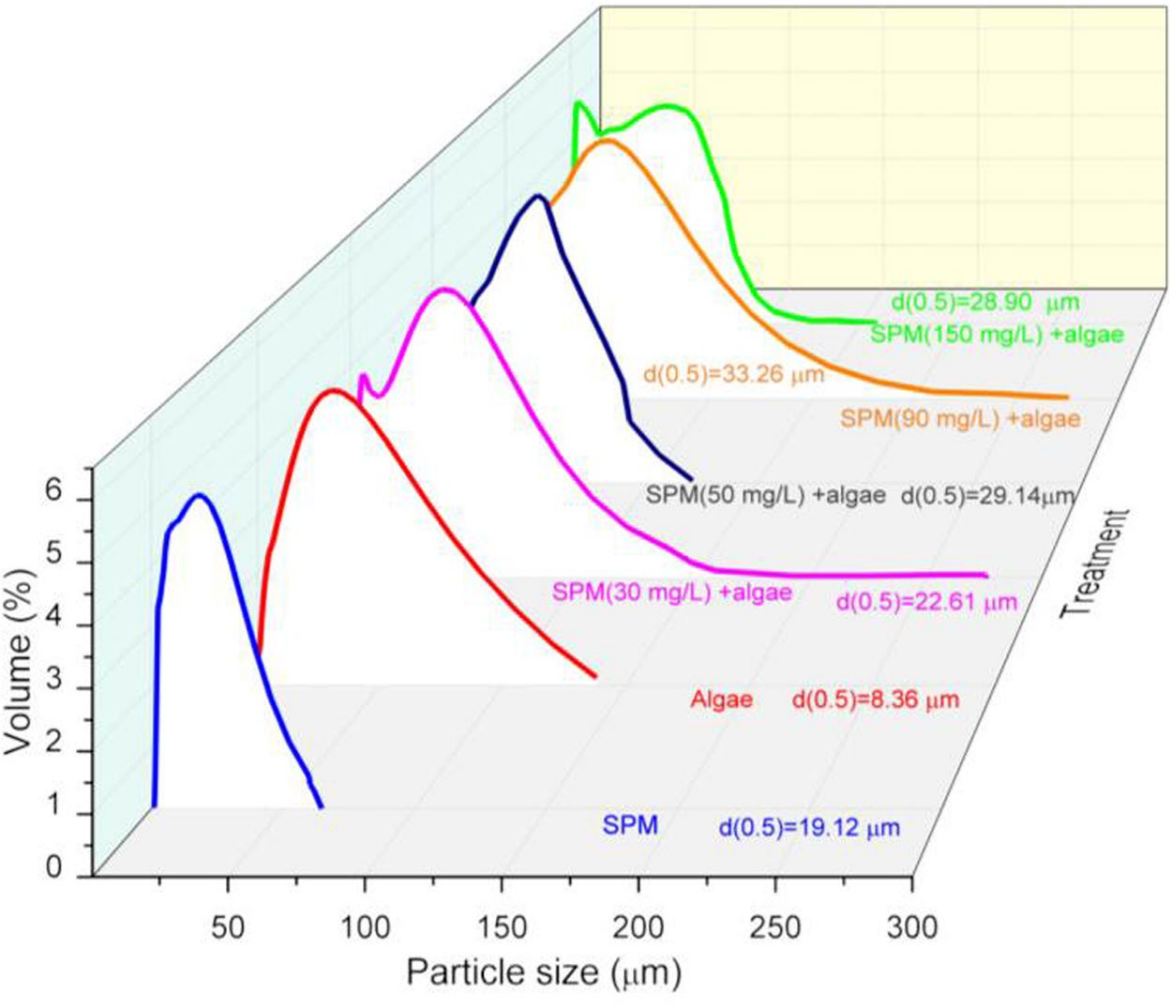
The size algae aggregation under varied SPM dosage.

Influence of SPM on algae aggregation. In this study, particle sizes of SPM and *Chlorella pyrenoidosa* were 19.1 and 8.3 μm, respectively. However, when SPM (at the dosage of 30 mg/L) was added to the algae solution, the size of the algae aggregates increased by approximately 15.7% compared to the size of SPM and 1.7-fold compared to the size of the algae aggregates (Fig. 4). Moreover, algae aggregate size significantly differed with SPM dosage. An increase in SPM dosage from 30 to 90 mg/L resulted in an increase in algae aggregation size by 47.1% (from 22.61 to 33.26 μm). However, with increasing SPM dosage up to 150 mg/L, algae aggregation size decreased (28.89 μm). When SPM and *Chlorella pyrenoidosa* were co-incubated, algae aggregation size increased by 7.4 to 22%.

**Figure 5:**
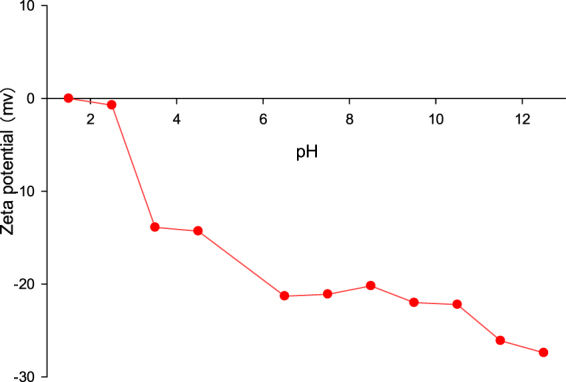
Surface charge changes of SPM. The data are the mean value of triplicates.

Surface charge of SPM. SPM values were mainly electro-negative in the pH range occurring in their natural environment. The isoelectric point of SPM was at pH 1.5 (Fig. 5). The zeta potential of SPM substantially declined, but was relatively stable and remained at about −20 mV in a pH range between 6.5 and 10.0, it then decreased to approximately −27 mv at pH 12.5. Under most natural water conditions, SPM is therefore net positively charged.

**Figure 6:**
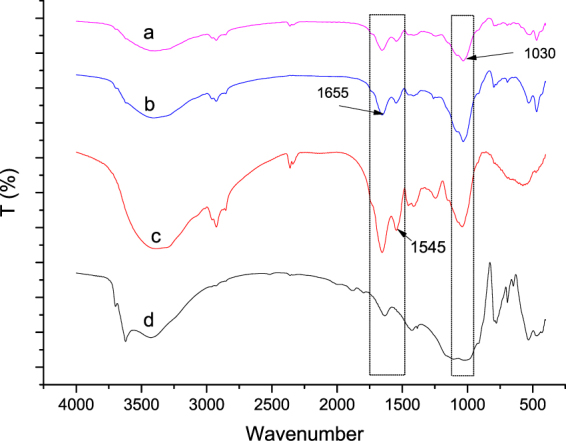
FTIR spectrum of SPM (**a**), *Chlorella pyrenoidosa* (**b**), SPM (30 mg/L) + *Chlorella pyrenoidosa* (**c**) and SPM (90 mg/L) + *Chlorella pyrenoidosa* (**d**) at pH 7.

Induction of functional groups on the algae cell surface by SPM. The FTIR spectra of the suspensions of *Chlorella pyrenoidosa*, SPM, and SPM (in varying dosages) plus *Chlorella pyrenoidosa* are shown in Fig. 6. For *Chlorella pyrenoidosa* (Fig. 6b), the spectral region from 1,000 to 1,200 cm^−1^ was dominated by the stretching (C–O–C) vibrations of polysaccharides (glycogen), with prominent features derived from carbohydrate adsorption bands at approximately 1,030 cm^−1^ for the C–O bonds included in polysaccharides. In the spectral region varying from 1,200 to 1,700 cm^−1^ for *Chlorella pyrenoidosa*, two absorbances at approximately 1,545 and 1,655 cm^−1^ (C=bond) could be related to the amide I group and the amide II group, respectively. In the spectral region varying from 3,000 to 3,500 cm^−1^, one prominent absorbance at approximately 3,450 cm^−1^ could be related to –OH or –NH (3,446–3,460 cm^−1^). With respect to SPM, the FTIR spectrum was featured at three absorbance at approximately 1,030, 1,545, and 1,655 cm^−1^ (Fig. 6a). When SPM was added, the absorbance at 1,655 cm^−1^ (C=bond) was largely depressed with increasing SPM dosage from 30 (Fig. 6c) to 90 mg/L (Fig. 6d). A similar pattern was observed for the absorbance at 3,450 cm^−1^.

## Discussion

Multiple factors, and especially nutrients concentration and water velocity, influenced algae density in the waterways. Phytoplankton density is generally not caused by a single environmental driver, but rather by multiple factors occurring simultaneously. To date, the factors identified as contributing towards the phytoplankton density included increased nutrient inputs, the transportation of cells or cysts via anthropogenic activities^1^. In addition, increased aquaculture production and/or overfishing, resulting in alteration of food webs, may also permit harmful species to dominate algal communities^12^. In the context of a changing climate, increased surface water temperatures can play a potential role in proliferation of phytoplankton species^13^. In the present study, using the recorded data, relationships between nutrient concentrations and phytoplankton density was illustrated in Fig. 1, of which the PCA analysis revealed that in the three experimental sites, NH_3_-N, NO_3_-N and PO_4_-P, TP and TN largely influenced the phytoplankton density, this finding was in agreement with the results of a previous study on the relationship between nutrient concentrations and phytoplankton density in the region around the Yangze River, including its estuary adjacent waters^14^. Water velocity was another factor that influencing phytoplankton density, which not only determined algae location in the water column, but also increase the advective transport of nutrients to algal cells^15^. In Yulin River, the water velocity showed distinct variations in response to TGR operations (Fig. S1), hence cause the suspension of sediment and increase of SPM content. It was widely accepted that phytoplankton density in a particular ecosystem depended on the extent to which different environmental variables played the key role. Nutrients were the dominate contributor for the proliferation of phytoplankton, however, was impacted by a variety of factors. In our study, despite the anthropogenic activities that led to the increase of nutrients, the co-existence between SPM and algae also impacted nutrient levels. Similarly, a previous study has found significantly higher SPM concentrations in the TGR than in the tributary rivers, such an imbalance could lead to lower phytoplankton density in the reservoir^10^.

Light attenuation and nutrients adsorption by SPM is responsible for algae density. SPM refers to all suspended particles, organic and inorganic, in aquatic ecosystems^16^. In river systems, the SPM content was largely impacted by the water velocity, which enhanced the suspension of sediment. Aimed at elucidating the contribution of SPM on the development phytoplankton communities, numerous studies had been conducted. It had been previously found that that the decrease of SPM concentrations in the water column with a concomitant increase in the penetration of solar radiation seems to be one of the main causes for the development of the phytoplankton in the Bahía Blanca Estuary^17^. This was supported by the study regarding phytoplankton composition, in the Guadiana estuary (SW Iberia) after dam construction^18^. The available references extended the understanding of to what extent the temporal variations of SPM regulated the phytoplankton by light-limitation, while the chemical and biological aspect of SPM and phytoplankton was deficient. In the present study, there was a significant negative correlation between the concentration of SPM and illumination intensity (Fig. 2), this finding was in agreement with the results of previous studies stating that certain concentrations of SPM could result in the extinction of light, thereby causing interspecific competition in phytoplankton^10^. The adsorption of dissolved phosphate onto SPM was considered as the key process buffering bioavailable phosphate concentrations. It was also found that a high portion of the P input into the Yellow river, China, was removed by adsorption, which was due to the high SPM values. In our study, the K_f_ of PO_4_-P on SPM was 0.8360 mg/L (Table 1), which was almost 50% of the P adsorption capability of SPM discovered previously^19^ and was remarkably lower than that in a heavy eutrophic water system in Suzhou, China^20^. We therefore suggested that the SPM in the Yulin River had a relatively weak P sorption capacity (Fig. 3), resulting in low P removal from the overlying water. Nutrients adsorption by SPM was one potential way. With respect to the adsorption of NH_3_-N, NO_3_-N, the studied SPM exhibited K_f_ values of 0.9026 and 0.6049, respectively, which were substantially lower compared to the references^21^. In terms of combined adsorption of nitrogen and phosphorus, we concluded that although the SPM could effectively impact phytoplankton growth by adsorbing or releasing nutrients, the reduction of nutrients by SPM may not play a key role in phytoplankton density. This finding was in contrast with the results of a previous study who observed that the nutrients for phytoplankton growth mainly originated from the releasing of particulate nutrients^22^. Moreover, the high concentrations of SPM may not only result in low photosynthesis and growth inhibition since light intensity was imperative for algal photosynthesis and growth, but also reduce the amounts of nutrients available for algal growth.

SPM and algal EPS induced co-aggregation. Under normal conditions, *Chlorella pyrenoidosa* had a size of approximately 8 μm. However, with the addition of SPM to the algae solution, algae size increased significantly and algae aggregation was observed, this effect was manifested when the SPM dosage increased from 30 to 90 mg/L (Fig. 4). In 2006, Pan and his colleges have evaluated the potential of soil/clays for cyanobacteria flocculation and found that electrostatic neutralization, netting, and bridging effects played dominated roles^23^. In our study, the Zeta potential of SPM was negatively charged and remained relatively stable at nearly −35 mv in wide pH range of 6.5–12.5 (Fig. 5). As *Chlorella pyrenoidosa* tends to float in water because of its negatively charged cell surface^1^, suggesting a low affinity to SPM. We therefore conclude that electrostatic neutralization may not be contribute significantly to the aggregation between SPM and *Chlorella pyrenoidosa* cells.

In a recent work, it had found that cyanobacterial EPS contributed to the aggregation kinetics of inorganic colloids in eutrophic shallow lakes, confirming the interaction between SPM and phytoplankton^11^. In our study, although the electrostatic neutralization as well as the netting and bridging effect of SPM can be neglected, we observed the growth of algae flocs, indicating additional mechanisms. With increased SPM dosage, amide II group (1,655 cm^−1^) and –OH or –NH (3,450 cm^−1^) were substantially decreased (Fig. 6), mainly because of the large amounts of functional groups of polysaccharides and proteins in the EPS, which may interact with SPM. Increased peaks at 1,039 cm^−1^ indicated that the functional groups of polysaccharides increased as a response to SPM addition.

**Figure 7:**
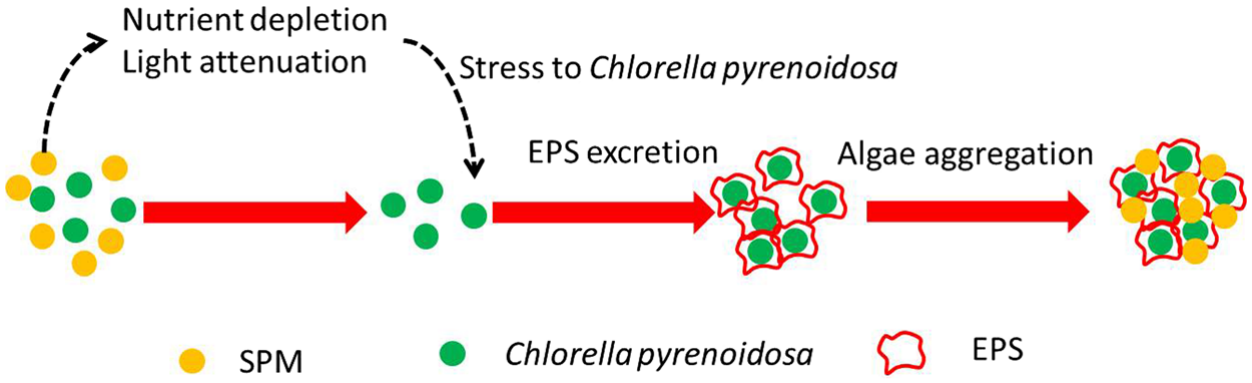
The mechanism model for *Chlorella pyrenoidosa* aggregation under the influence of SPM.

Cyanobacterial EPS could induce aggregation of inorganic colloids in eutrophic shallow lakes^11^. The EPS, mainly a result of secretion and excretion when the phytoplankton cells was under stress, can significantly influence the physicochemical properties and aggregation potential of phytoplankton. In a previous study, inorganic phosphorus depletion resulted in increased phytoplankton EPS^24^. Under flagellate grazing pressure, synthesis and secretion of EPS of *Microcystis aeruginosa* cells can also be prompted^25^. Other factors that induced EPS release from phytoplankton included light and temperature^26^. In this study, the depleted nutrients and depressed light intensity, caused by SPM, possibly triggered the release of EPS from *Chlorella pyrenoidosa*, as was illustrated in Fig. 7. However, since the d(0.5) of the SPM in this study remained at 19.12 μm, the aggregation of *Chlorella pyrenoidosa* depressed as the effect was largely depended on particle size^11^.

Environmental implications for eutrophication control in TGR. SPM can potentially affect the growth of phytoplankton by adsorbing or releasing nutrients. In our study, however, nutrient adsorption on SPM was weak, indicating that the reduction of bioavailable nutrients was largely related to the high quantity of SPM in the Yulin River. Although a high concentration of SPM reduced nutrients for algal growth in the water column, SPM could reduce the underwater light intensity, which often results in decreased photosynthesis and growth inhibition^10^. While in many rivers, dams are removing SPM from the water column, it is important to note that the SPM in the Yangze River differs greatly due to the different hydraulic conditions since the construction of the TGD. Dam construction started in 1993, and closure of the reservoir and regulation of the water level started in 2003. Since then, the annual mean SPM load at the Yangtze River mouth (Datong Hydrological Station) has decreased by 23.4% in 2007^27^, the similar trend was also found in a recent study that the annual mean total suspended matter (TSM) in the Yangtze estuary showed a significant decreasing trend within 11 years (−2.8 mg L^−1^ yr^−1^)^28^. Nevertheless, under the joint effects driven by SPM, although harmful algae blooms (HABs) frequently occurred in tributaries of the TGR, they could be kept under control. A comprehensive review of available evidence suggest that phytoplankton are known to be sensitive to environmental change, and both the nutrients and SPM are among the key environmental drivers whose content is predicted to continue changing in TGR in the future, this indicate that if the SPM is to be further reduced, the dissolved nutrients in the tributaries of the TGR will be further increased, hence the concern of SPM content should be taken into consideration in ecological operation in order to prevent severe and irreversible water quality problems in this large ecological system. Therefore, understanding the roles of multiple factors on phytoplankton density, including the individual and interactive effects of SPM and phytoplankton, will greatly assist TGR operators as they seek to mitigate these increasingly common and problematic HABs in tributary and in the reservoir itself.

In this study, we confirmed the overarching hypothesis that SPM impacts phytoplankton density through interactive effects, including nutrient adsorption, light attenuation, and the interaction between the EPS of *Chlorella pyrenoidosa* (Fig. 7). However, it was hard to discriminate the dominated approach that impact the phytoplankton density according to the available data, which needs further study in laboratory condition. Another issue that needs to address is that the impact of SPM on phytoplankton may not only associate with the SPM load, but also depend on the characteristics of the SPM, including the surface area, the distribution of SPM size, surface morphology, or surface fractal dimension. Therefore, the influences of SPM characteristics on nutrient adsorption or algae aggregation need to be further assessed.

## Material and Methods

### Study area

**Figure 8:**
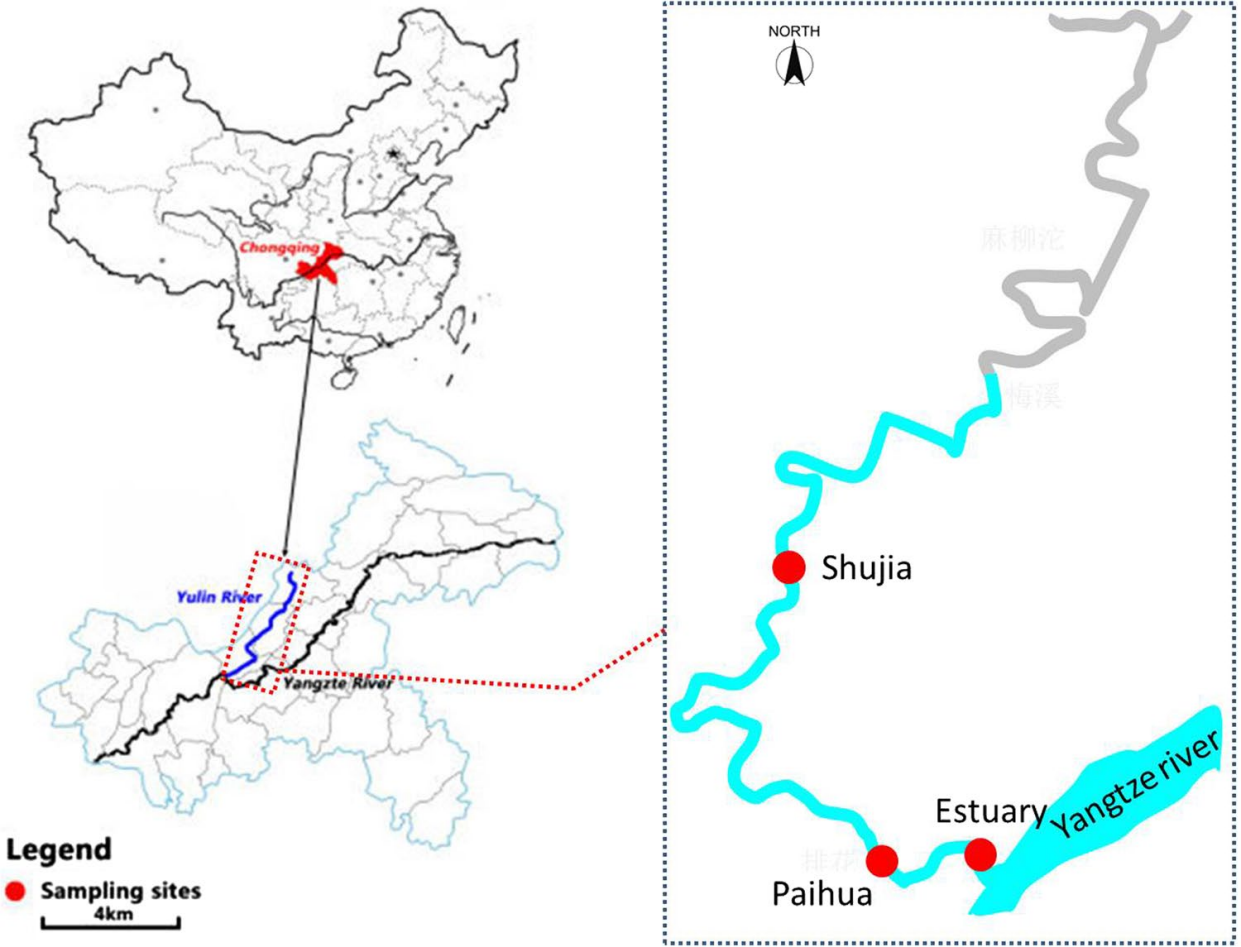
Geographic locations of Yulin River and sampling sites. The map is generated using ArcGIS (version 10.2).

The Yulin River (106°27′30″~106°57′58″E, 29°34′45″~30°07′22″N), one of the tributary rivers in the higher reaches of the TGR, originates in the Gongqiaoba Reservior, which is located in the southeastern part of Sichuan Province, China. It is about 218.2 km long and has a total watershed area of 3,861 km^2^. After the initial impoundment of the TGR in 2003, the Yulin River annually changes from river-type (from April to October) to lake-type reservoir (from October to March). The backwater extended to about 40 km when the reservoir was filled to the normal water levels (175 m). The flow pattern of the backwater zone substantially differ between the impoundment period and the drain period, which usually leads to precipitation and resuspension of the SPM. In addition, since the impoundment of the TGR, algal blooms have been occurring frequently in the Yulin River. In this study, sampling was conducted in the estuary, Paihua (PH), and Shujia (SJ) sites which were affected by the backwater of the Yangtze River. Figure 8 shows the three sampling sites in the Yulin River.

### Sample collection and preparation

Sampling was conducted monthly from January 2013 to December 2014. The water level stages of the TGR varies annually, from September to April, the reservoir is impounded and from May to August, water is being discharged.

We measured water temperature and DO *in-situ* with a portable meter YSI-556 (YSI, USA), in addition to water depth and transparency (Secchi disk depth). Photosynthetically available radiation (PAR) along the depth was measured with an underwater illuminometer (IL1400A, China). Concentrations of total nitrogen (TN), ammonia nitrogen (NH_3_-N), nitrate nitrogen (NO_3_-N), total phosphorus (TP), and dissolved phosphates (PO_4_-P) in each layer were measured spectrometrically (SHIMADZU-UV-3600, Japan)^29^. The SPM samples were also assessed by filtering the water samples using a peristaltic pump (80EL005, Millipore Co., USA) and a filter plate with the pore size of 0.45-μm glass fiber filters (GFFs). The corresponding GFFs were air-dried until constant weight and the SPM contents were determined by gravimetric analysis. All parameters were measured along the entire depth (differ with TGR operation and maximally reach 22 m) in 2-m intervals.

### Algae species and reagents

The dominant phytoplankton species in the Yulin River, *Chlorella pyrenoidosa*, was obtained from the Freshwater Algae Culture Collection (FACHB-5) at the Institute of Hydrobiology (FACHB-Collection, Wuhan, China). Algae were cultivated in an illuminating incubator (LRH-250-G, China) with continuous cool white fluorescent light of 2 000–3 000 lux on a 12 h light and 12 h darkness regime; temperature was maintained at 25 ± 1 °C^30^. All reagents used in this study were of analytical grade.

### Light attenuation by SPM under varied water velocity

Light attenuation in water containing SPM was evaluated in mesocosm scales. Five groups of columns with a diameter of 20 cm and a depth of 80 cm were used. To each column, we added 0.48 g SPM collected from the Yulin River. The samples were mixed with an electric blender and a water flow of 0.1, 0.2, 0.4, 0.6, and 0.8 m/s, respectively, was applied. When mixing was stable, we measured illumination density at 10-cm intervals using a PAR. Values of SPM were measured corresponding to the depth.

### Batch adsorption experiment

The batch adsorption experiments were conducted in order to evaluate the adsorption kinetics and of the impacts of dissolved nutrients (NH_3_-N, NO_3_-N and PO_4_-P) on SPM. All batch adsorption experiments were performed according to the following procedures: 35 mL of dissolved nutrients and 200 mg/L of SPM were added into 50-mL centrifuge tubes. Subsequently, the centrifuge tubes were shaken on a rotary shaker at 30 rpm. Blanks containing no suspended particulate matter were included in each experiment. To study the impact of pH on nutrient adsorption, we added 0.2 g of the adsorbent containing 50 ml dissolved nutrient solution. The pH of the solutions was adjusted using 0.1 M HCl or NaOH solution to the desired values (2.50 ± 0.01, 3.50 ± 0.01, 4.50 ± 0.01, 5.50 ± 0.01, 7.00 ± 0.01, 9.00 ± 0.01, and 11.00 ± 0.01). When the equilibrium time and the optimal pH condition were obtained, the adsorption capacity of NH_3_-N, NO_3_-N, and PO_4_-P on SPM was then determined as described below. The NH_3_-N, NO_3_-N, and PO_4_-P adsorption capabilities of SPM were tested under the following initial nutrient concentrations: i) 0.1, 0.2, 0.5, 0.8, 1.0, 1.5, 2.0 mg/L NH_3_-N, using NH_4_Cl in distilled water, ii) 0.5, 1.0, 1.5, 2.0, 3.0, 5.0, 7.0 mg/L NO_3_-N, using KNO_3_, and iii) 0.1, 0.2, 0.5, 0.8, 1, 1.5, 2.5 mg/L PO_4_-P, using KH_2_PO_4_. Each test employed 0.2 g of SPM (three control groups: NH_3_-N, NO_3_-N, and PO_4_-P solutions without SPM) in 100-mL glass Erlenmeyer flasks containing 50 mL of NH_3_-N, NO_3_-N, and PO_4_-P solution, respectively. Two drops of chloroform were added to each sample to inhibit microbial growth. The Erlenmeyer flasks were continuously shaken (170 rpm) on a rotary shaker (KASI KSI-200L, Korea) for 24 h. Subsequently, the samples were centrifuged at 4,000 rpm g for 5 min and an aliquot of the supernatant was filtered through Whatman GF/C filters (0.45 μm) and analyzed for NH_3_-N, NO_3_-N, and PO_4_-P^29^. All experiments were conducted in triplicate. The temperature during the batch adsorption experiment was maintained at 25 ± 1 °C. Adsorption behavior was interpreted using the most commonly used adsorption equations, including Freundlich and Langmuir sorption isotherms^31^.

To determine the amounts of adsorbed nutrients onto the SPM particles, we calculated the differences between the initial nutrient concentrations and the equilibrium concentrations. Freundlich and Langmuir sorption isotherms were generated by fitting the adsorption data under various concentrations to determine the adsorption capacity of SPM. The Freundlich isotherm was expressed as the following equation:

1 $$Cs={K}_{f}\times {C}_{e}^{n}$$

The Langmuir equation, in its original form, can be expressed as:

2 $${\rm{Cs}}=\frac{{K}_{1}\times \mathrm{Qmax}\,\times {\rm{Ce}}}{1+{K}_{1}\times {\rm{Ce}}}$$

where Cs (mg/g) is the amount of nutrients adsorbed on to the SPM, Ce (mg/L) is the concentration of nutrients in the supernatant, K_f_ (mg/L) is the Freundlich adsorption coefficient, n is a Freundlich constant parameter describing the degree of nonlinearity, K_l_ (mg/L) is the Langmuir adsorption coefficient and is a constant related to the adsorption affinity, Qmax (mg/L) is the maximum sorbent loading. All adsorption data were plotted for nonlinear isotherms and the regression coefficients were calculated.

### Algal aggregation

Based on the results of the PCA analysis, SPM from the Paihua site was used in the algal flocculation test. The experiments were performed in a jar test apparatus (ZR3-6, Zhongrun Water Industry Technology Development Co. Ltd., China) with a series of 300-ml beakers containing 200 ml of *Chlorella pyrenoidosa* cultures in mid exponential growth phase. Initial *Chlorella pyrenoidosa* abundance was 4.5 × 10^6 ^cells/mL. After addition of the SPM (at 30, 50, 90, and 150 mg/L, representing the concentrations of SPM in the Paihua site during the discharging period throughout the field measurement), the solution was stirred at 200 rpm for 1 min and at 40 rpm for another 15 min. The control was run in the above-mentioned algae media without the addition of SPM. After sedimentation for 30 min, samples were collected from 5 cm below the surface for flocs size determination. All flocculation experiments were conducted in triplicate, the results are presented as mean values and standard deviations. Surface charge of SPM was characterized using a Zetasizer 2000 (Malvern Co. United Kingdom). Algal floc size was quantitatively assessed using a laser particle size analyzer Mastersizer 2000 (Malvern Co. United Kingdom) and denoted by the measured mean diameter (D_0.5_)^31^.

### Statistical analyses

We used principal components analysis (PCA) to interpret the main factors of phytoplankton abundance. The ANOVA was used to determine significant differences between measurements, with a significance threshold set at P < 0.05.

## Electronic supplementary material

Fig S1 and Table S1

## References

[1] Davidson K (2012). Harmful algal blooms: How strong is the evidence that nutrient ratios and forms influence their occurrence?. Estuarine, Coastal and Shelf Science.

[2] Yang C (2016). A comprehensive insight into functional profiles of free-living microbial community responses to a toxic Akashiwo sanguinea bloom. Scientific Reports.

[3] Conley DJ (2009). Controlling Eutrophication: Nitrogen and Phosphorus. Science.

[4] Yin H (2017). Interactions of riverine suspended particulate matter with phosphorus inactivation agents across sediment-water interface and the implications for eutrophic lake restoration. Chemical Engineering Journal.

[5] Liu SM (2015). Response of nutrient transports to water–sediment regulation events in the Huanghe basin and its impact on the biogeochemistry of the Bohai. Journal of Marine Systems.

[6] López-Tarazón JA (2016). Suspended sediment, carbon and nitrogen transport in a regulated Pyrenean river. Science of The Total Environment.

[7] Zeng H (2006). Distribution of phytoplankton in the Three-Gorge Reservoir during rainy and dry seasons. Science of The Total Environment.

[8] Xu Y (2011). Asynchrony of spring phytoplankton response to temperature driver within a spatial heterogeneity bay of Three-Gorges Reservoir, China. Limnologica - Ecology and Management of Inland Waters.

[9] Middelburg JJ, Herman PMJ (2007). Organic matter processing in tidal estuaries. Marine Chemistry.

[10] Gao M (2016). Spatiotemporal patterns of surface-suspended particulate matter in the Three Gorges Reservoir. Environmental Science and Pollution Research.

[11] Xu H (2016). Aggregation kinetics of inorganic colloids in eutrophic shallow lakes: Influence of cyanobacterial extracellular polymeric substances and electrolyte cations. Water Research.

[12] Heisler J (2008). Eutrophication and harmful algal blooms: A scientific consensus. Harmful Algae.

[13] Paerl HW, Huisman J (2008). Blooms like it hot. Science.

[14] Chai C (2006). The Status and Characteristics of Eutrophication in the Yangtze River (Changjiang) Estuary and the Adjacent East China Sea, China. Hydrobiologia.

[15] Warnaars TA, Hondzo M (2006). Small-scale fluid motion mediates growth and nutrient uptake of Selenastrum capricornutum. Freshwater Biology.

[16] Waters, T. F. Sediments in Streams: Sources, Biological Effects and Controls. *American Fisheries Society* (1995).

[17] Guinder VA (2009). Particulate suspended matter concentrations in the Bahía Blanca Estuary, Argentina: Implication for the development of phytoplankton blooms. Estuarine, Coastal and Shelf Science.

[18] Domingues RB (2012). Phytoplankton composition, growth and production in the Guadiana estuary (SW Iberia): Unraveling changes induced after dam construction. Science of The Total Environment.

[19] Xiao, Y. *et al*. SurIIace properties of sediments and its effect on phosphorus adsorption. *Joumal of Sediment Research* **6**, 64–68 (2011).

[20] Li D, Huang Y (2013). Phosphorus uptake by suspended sediments from a heavy eutrophic and standing water system in Suzhou, China. Ecological Engineering.

[21] Xia X (2017). Enhanced nitrogen loss from rivers through coupled nitrification-denitrification caused by suspended sediment. Science of The Total Environment.

[22] Jones JR, Knowlton MF (2005). Suspended solids in Missouri reservoirs in relation to catchment features and internal processes. Water Research.

[23] Zou H (2006). Removal of cyanobacterial blooms in Taihu Lake using local soils II. Effective removal of Microcystis aeruginosa using local soils and sediments modified by chitosan. Environmental Pollution.

[24] Urbani R (2005). Extracellular carbohydrates released by the marine diatoms Cylindrotheca closterium, Thalassiosira pseudonana and Skeletonema costatum: Effect of P-depletion and growth status. Science of The Total Environment.

[25] Yang Z (2008). Changes in the morphology and polysaccharide content of Microcystis aeruginosa (cyanobacteria) during flagellate grazing. Journal of Phycology.

[26] Mishra A, Jha B (2009). Isolation and characterization of extracellular polymeric substances from micro-algae Dunaliella salina under salt stress. Bioresource Technology.

[27] Koshikawa MK (2007). Distributions of dissolved and particulate elements in the Yangtze estuary in 1997–2002: Background data before the closure of the Three Gorges Dam. Estuarine, Coastal and Shelf Science.

[28] Feng L (2014). Influence of the Three Gorges Dam on total suspended matters in the Yangtze Estuary and its adjacent coastal waters: Observations from MODIS. Remote Sensing of Environment.

[29] APHA-AWWA-WEF. Standard methods for the examination of water and wastewater (21st ed.). *Washington, DC: American Public Health Association* (2005).

[30] Shi W (2016). Removal of Microcystis aeruginosa using cationic starch modified soils. Water Research.

[31] Park JH (2015). Evaluation of phosphorus adsorption capacity of sesame straw biochar on aqueous solution: influence of activation methods and pyrolysis temperatures. Environmental Geochemistry and Health.

