# Supplementary material for: New insights into the impacts of suspended particulate matter on phytoplankton density in a tributary of the Three Gorges Reservoir, China
Source: Sci Rep. 2017 Oct 18;7:13518. doi: 10.1038/s41598-017-13235-0 (PMC5647362; doi:10.1038/s41598-017-13235-0)
Supplement: Supplementary file 1 — Fig S1 and Table S1 [file 41598_2017_13235_MOESM1_ESM.doc]

***Supporting information***

New insights into the impacts of suspended particulate matter on phytoplankton density in a tributary of the Three Gorges Reservoir, China

Qiang He ^1^, Yixi Qiu ^1^, Haohang Liu ^1^, Xingfu Sun ^2^, Li Kang ^1^, Li Cao ^3^, Hong Li ^1^, Hainan Ai ^1*^

^1^ Key Laboratory of Eco-Environment of Three Gorges Region, Ministry of Education, Chongqing University, Chongqing 400044, China

^2^ Xiamen Municipal Engineering Design Institute CO., LTD, Chongqing 401122, China

^3^ Chongqing Green Environment Protection Technology CO., LTD, Chongqing 400045, China

Fig. S1 The variation of water velocity during the study periods

Table S1 Nutrients and chla variations in Yulin River during 2013-2014

| **date** | **Sampling site** | **TP**  **（**mg/L**）** | **TN**  **（**mg/L**）** | PO_4_-P**（**mg/L**）** | NO_3_-N**（**mg/L**）** | NH_3_-N**（**mg/L**）** | Chla**（**mg/m^3^**）** |
| --- | --- | --- | --- | --- | --- | --- | --- |
| 2013/1/21 | Estuary | 0.14±0.01 | 2.28±0.08 | 0.03±0.01 | 1.68±0.05 | 0.25±0.01 | 1.40±0.05 |
|  | Paihua | 0.13±0.01 | 2.16±0.09 | 0.08±0.01 | 1.67±0.09 | 0.14±0.01 | 27.50±0.15 |
|  | Shujia | 0.11±0.01 | 2.87±0.11 | 0.03±0.01 | 2.15±0.10 | 0.38±0.01 | 6.60±0.23 |
| 2013/2/22 | Estuary | 0.15±0.01 | 2.46±0.05 | 0.10±0.01 | 1.19±0.03 | 0.64±0.01 | 1.40±0.20 |
|  | Paihua | 0.12±0.01 | 2.78±0.03 | 0.04±0.01 | 2.27±0.03 | 0.18±0.01 | 1.80±0.04 |
|  | Shujia | 0.13±0.01 | 2.43±0.06 | 0.02±0.01 | 2.05±0.06 | 0.15±0.01 | 0.25±0.01 |
| 2013/3/22 | Estuary | 0.02±0.001 | 1.37±0.01 | 0.01±0.001 | 1.00±0.01 | 0.31±0.01 | 8.80±0.08 |
|  | Paihua | 0.02±0.001 | 1.42±0.01 | 0.01±0.001 | 1.10±0.01 | 0.18±0.01 | 3.20±0.03 |
|  | Shujia | 0.03±0.001 | 1.37±0.01 | 0.07±0.01 | 1.01±0.01 | 0.09±0.00 | 9.90±0.26 |
| 2013/4/25 | Estuary | 0.02±0.001 | 1.69±0.01 | 0.01±0.001 | 0.85±0.01 | 0.24±0.01 | 10.60±0.10 |
|  | Paihua | 0.04±0.001 | 1.67±0.01 | 0.01±0.001 | 0.99±0.02 | 0.15±0.01 | 2.00±0.05 |
|  | Shujia | 0.04±0.00 | 2.03±0.01 | 0.02±0.001 | 0.84±0.01 | 0.56±0.01 | 10.90±0.22 |
| 2013/5/24 | Estuary | 0.03±0.001 | 1.60±0.01 | 0.01±0.001 | 0.62±0.011 | 0.31±0.006 | 8.25±0.22 |
|  | Paihua | 0.04±0.001 | 1.61±0.01 | 0.02±0.000 | 0.61±0.01 | 0.35±0.02 | 8.05±0.33 |
|  | Shujia | 0.03±0.001 | 1.72±0.01 | 0.01±0.001 | 0.64±0.02 | 0.31±0.011 | 9.10±0.54 |
| 2013/6/21 | Estuary | 0.03±0.002 | 2.87±0.07 | 0.01±0.001 | 2.30±0.18 | 0.16±0.01 | 4.70±0.33 |
|  | Paihua | 0.02±0.001 | 3.22±0.05 | 0.01±0.000 | 0.35±0.02 | 0.55±0.03 | 5.10±0.28 |
|  | Shujia | 0.04±0.001 | 2.08±0.02 | 0.01±0.00 | 1. 15±0.01 | 0.54±0.02 | 9.10±0.88 |
| 2013/7/25 | Estuary | 0.06±0.001 | 2.32±0.04 | 0.01±0.001 | 1.61±0.05 | 0.24±0.00 | 9.60±0.06 |
|  | Paihua | 0.05±0.001 | 2.21±0.06 | 0.01±0.001 | 1.62±0.07 | 0.31±0.01 | 11.1±0.33 |
|  | Shujia | 0.05±0.001 | 1.96±0.06 | 0.01±0.000 | 1.27±0.02 | 0.31±0.01 | 9.50±0.08 |
| 2013/8/23 | Estuary | 0.04±0.001 | 1.76±0.001 | 0.01±0.00 | 1.13±0.07 | 0.22±0.01 | 16.00±0.52 |
|  | Paihua | 0.04±0.001 | 1.82±0.010 | 0.02±0.001 | 0.21±0.002 | 0.69±0.03 | 11.20±0.27 |
|  | Shujia | 0.03±0.001 | 1.70±0.01 | 0.01±0.00 | 0.19±0.001 | 0.41±0.01 | 11.10±0.72 |
| 2013/9/23 | Estuary | 0.02±0.001 | 1.55±0.01 | 0.02±0.001 | 1.10±0.02 | 0.27±0.01 | 4.80±0.21 |
|  | Paihua | 0.03±0.001 | 1.46±0.06 | 0.01±0.001 | 1.08±0.02 | 0.22±0.01 | 9.40±0.16 |
|  | Shujia | 0.05±0.001 | 1.75±0.05 | 0.03±0.001 | 1.22±0.08 | 0.33±0.01 | 3.50±0.15 |
| 2013/10/25 | Estuary | 0.11±0.00 | 1.61±0.03 | 0.05±0.00 | 1.08±0.02 | 0.25±0.01 | 11.00±0.51 |
|  | Paihua | 0.09±0.00 | 1.55±0.04 | 0.07±0.00 | 0.78±0.01 | 0.40±0.00 | 3.90±0.05 |
|  | Shujia | 0.24±0.002 | 1.51±0.06 | 0.01±0.001 | 1.09±0.08 | 0.17±0.01 | 2.50±0.06 |
| 2013/11/23 | Estuary | 0.13±0.00 | 1.78±0.05 | 0.08±0.00 | 1.22±0.09 | 0.19±0.00 | 0.658±0.012 |
|  | Paihua | 0.09±0.00 | 1.68±0.08 | 0.06±0.00 | 1.07±0.02 | 0.42±0.01 | 0.97±0.01 |
|  | Shujia | 0.09±0.00 | 1.75±0.04 | 0.05±0.00 | 1.25±0.03 | 0.22±0.00 | 1.38±0.02 |
| 2013/12/21 | Estuary | 0.20±0.01 | 1.91±0.06 | 0.05±0.00 | 1.47±0.09 | 0.11±0.01 | 0.893±0.010 |
|  | Paihua | 0.15±0.01 | 2.15±0.16 | 0.04±0.00 | 1.58±0.04 | 0.09±0.01 | 8.12±0.05 |
|  | Shujia | 0.12±0.01 | 1.98±0.05 | 0.03±0.00 | 1.35±0.04 | 0.28±0.01 | － |
| 2014/1/11 | Estuary | 0.28±0.01 | 1.64±0.13 | 0.10±0.00 | 1.29±0.06 | 0.15±0.01 | 0.49±0.01 |
|  | Paihua | 0.23±0.01 | 1.83±0.08 | 0.10±0.01 | 1.35±0.02 | 0.22±0.01 | 3.249±0.015 |
|  | Shujia | 0.11±0.01 | 1.94±0.12 | 0.06±0.00 | 1.12±0.03 | 0.21±0.01 | 2.346±0.016 |
| 2014/2/22 | Estuary | 0.21±0.01 | 1.55±0.10 | 0.09±0.00 | 1.11±0.02 | 0.19±0.01 | 16.50±0.51 |
|  | Paihua | 0.24±0.01 | 1.86±0.08 | 0.12±0.01 | 1.25±0.09 | 0.16±0.01 | 14.40±0.21 |
|  | Shujia | 0.13±0.01 | 1.93±0.06 | 0.05±0.00 | 1.15±0.09 | 0.13±0.01 | 9.50±0.28 |
| 2014/3/23 | Estuary | 0.39±0.01 | 3.31±0.02 | 0.07±0.00 | 2.09±0.05 | 0.50±0.03 | 18.70±0.24 |
|  | Paihua | 0.79±0.01 | 3.08±0.08 | 0.07±0.00 | 1.76±0.02 | 0.82±0.02 | 14.40±0.38 |
|  | Shujia | 0.48±0.01 | 3.58±0.06 | 0.11±0.01 | 2.99±0.05 | 0.49±0.05 | 9.50±0.18 |
| 2014/4/25 | Estuary | 0.09±0.00 | 2.24±0.03 | 0.02±0.00 | 1.45±0.02 | 0.17±0.01 | 2.60±0.06 |
|  | Paihua | 0.12±0.01 | 2.47±0.02 | 0.03±0.00 | 1.67±0.01 | 0.34±0.01 | 2.60±0.03 |
|  | Shujia | 0.11±0.00 | 2.47±0.09 | 0.05±0.00 | 1.44±0.05 | 0.33±0.01 | 2.70±0.05 |
| 2014/5/24 | Estuary | 0.07±0.00 | 2.75±0.02 | 0.02±0.00 | 2.24±0.03 | 0.33±0.01 | 0.80±0.02 |
|  | Paihua | 0.15±0.01 | 3.06±0.06 | 0.07±0.00 | 2.14±0.03 | 0.42±0.03 | 0.70±0.01 |
|  | Shujia | 0.09±0.00 | 3.19±0.06 | 0.05±0.00 | 2.36±0.02 | 0.30±0.00 | 0.70±0.01 |
| 2014/6/20 | Estuary | 0.10 | 2.70±0.06 | 0.07±0.00 | 1.45±0.02 | 0.53±0.01 | 0.80±0.01 |
|  | Paihua | 0.08±0.00 | 2.84±0.05 | 0.04±0.00 | 2.22±0.01 | 0.32±0.00 | 0.50±0.01 |
|  | Shujia | 0.08±0.00 | 2.57±0.005 | 0.05±0.00 | 2.14±0.02 | 0.12±0.00 | 0.90±0.02 |
| 2014/7/20 | Estuary | 0.04±0.00 | 1.94±0.03 | 0.01±0.000 | 092±0.00 | 0.15±0.00 | 0.80±0.02 |
|  | Paihua | 0.15±0.00 | 3.06±0.04 | 0.01±0.000 | 2.64±0.04 | 0.12±0.00 | 0.70±0.01 |
|  | Shujia | 0.02±0.00 | 1.83±0.03 | 0.01±0.000 | 1.02±0.00 | 0.19±0.00 | 2.30±0.05 |
| 2014/8/21 | Estuary | 0.12±0.00 | 1.82±0.01 | 0.07±0.00 | 1.05±0.01 | 0.24±0.00 | 0.50±0.01 |
|  | Paihua | 0.07±0.00 | 1.96±0.02 | 0.01±0.001 | 1.05±0.01 | 0.34±0.00 | 0.70±0.01 |
|  | Shujia | 0.17±0.00 | 1.98±0.02 | 0.06±0.001 | 1.19±0.02 | 0.46±0.00 | 0.60±0.01 |
| 2014/9/26 | Estuary | 0.12±0.01 | 2.24±0.02 | 0.01±0.000 | 1.56±0.01 | 0.42±0.01 | 1.20±0.03 |
|  | Paihua | 0.18±0.009 | 2.09±0.01 | 0.07±0.002 | 1.53±0.01 | 0.31±0.01 | 1.20±0.06 |
|  | Shujia | 0.20±0.01 | 2.34±0.01 | 0.07±0.002 | 1.49±0.06 | 0.24±0.01 | 1.30±0.05 |
| 2014/10/24 | Estuary | 0.19±0.001 | 1.99±0.016 | 0.07±0.001 | 1.33±0.011 | 0.28±0.00 | 0.70±0.01 |
|  | Paihua | 0.11±0.002 | 2.25±0.021 | 0.07±0.001 | 1.92±0.02 | 0.36±0.00 | 0.70±0.01 |
|  | Shujia | 0.11±0.001 | 1.86±0.018 | 0.04±0.001 | 0.04±0.00 | 0.27±0.00 | 1.00±0.01 |
| 2014/11/24 | Estuary | 0.18±0.001 | 2.20±0.01 | 0.07±0.004 | 1.62±0.001 | 0.28±0.01 | 0.70±0.01 |
|  | Paihua | 0.07±0.000 | 1.82±0.02 | 0.01±0.001 | 1.13±0.005 | 0.27±0.01 | 1.10±0.01 |
|  | Shujia | 0.07±0.001 | 1.62±0.06 | 0.01±0.000 | 1.22±0.008 | 0.17±0.01 | 1.60±0.01 |
| 2014/12/24 | Estuary | 0.27±0.001 | 1.81±0.002 | 0.13±0.001 | 1.36±0.02 | 0.15±0.01 | 0.40±0.01 |
|  | Paihua | 0.19±0.001 | 1.95±0.003 | 0.08±0.001 | 1.56±0.02 | 0.10±0.00 | 0.50±0.01 |
|  | Shujia | 0.10±0.001 | 1.87±0.006 | 0.04±0.00 | 1.29±0.02 | 0.22±0.01 | 1.60±0.02 |
